# Towards achieving the sustainable development goal 9: Analyzing the role of green innovation culture on market performance of Chinese SMEs

**DOI:** 10.3389/fpsyg.2022.1018915

**Published:** 2023-01-05

**Authors:** Changjing Wei, Xuesen Cai, Xiaowei Song

**Affiliations:** School of Management, Ocean University of China, Qingdao, Shandong, China

**Keywords:** green innovation, market performance, green innovation culture, marketing innovation, product innovation, small and medium-sized enterprises

## Abstract

Green innovation culture is essential to the Chinese 14th five-year plan aligned with sustainable development goal 9. This study examines the relationship between green innovation culture and market performance of Chinese small and medium-sized enterprises (SMEs). We evaluated hypothesis by taking a sample of 564 SMEs employees in China through an online survey. The structural equation modelling (SEM) method is used to analyze data. The findings showed that green innovation culture positively influence product and marketing innovation. Similarly, marketing innovation positively affects product innovation and market performance. In addition, product innovation has a substantial effect on market performance. The outcomes of this study imply that SMEs in emerging economies should concentrate on green innovation culture to improve their market performance. In addition, the identification of study limitations and suggestions for further research are also addressed for all stakeholders involved with SMEs.

## Introduction

Over the years, regulators across the globe have been eager to set legislation and standards for green goods and services (Chai et al., 2021; Su et al., 2021). Due to increased industrialization, environmental concerns are becoming a significant concern for corporations, governments, and society (Cabral and Lochan Dhar, 2019; Yang et al., 2021). Various external causes pressure corporations to pay heightened attention to environmental management (Jia et al., 2021; Hao et al., 2022). One body of literature on sustainable development demonstrates that companies adopt green practices in response to societal and legitimate pressures; whereas another body of literature emphasizes that being pro-environmental brings substantial advantages, including assisting in increasing productive capacity and lowering costs, nature stewardship, and creating a favorable impression of the company (Qu et al., 2022; Xue et al., 2022).

Therefore, several businesses have acknowledged the significance of green innovation culture (GIC) and adopted it as a viable strategy for competitive advantage (Wong, 2017). China’s small and medium enterprises (SMEs) have seen a substantial shift over the past few decades (Zheng S. et al., 2022). Buyers’ knowledge and related legislation have generated a sense of urgency about environmental preservation. Before the advent of environment-related laws in many regions of the globe, the SMEs sector saw environmental management challenges as an unnecessary expenditure; some even viewed it as an impediment to the organization’s development (Doghan et al., 2022). This has resulted in several environmental problems (Wu et al., 2021; Ren et al., 2022). Additionally, legislation and ISO standards, restrictions on the consumption of various harmful chemicals, waste electrical and electronic equipment, and consumers’ sheer awareness of the environment make GIC a vital aspect of a business (Muisyo and Qin, 2021; Wang H. et al., 2021).

Prior research has shown that core skills are vital to enhancing organizational performance through knowledge-based initiatives to promote GIC (Shujahat et al., 2019; Chen et al., 2022). Additionally, GIC is the primary predictor of an organization’s viability and enhancing long-term financial success (Ahmad et al., 2021; Qu et al., 2022). GIC helps to transform and identify new opportunities, offering companies a competitive edge by enabling them to provide superior products and services for potential clients, dramatically influencing how organizations operate (Doghan et al., 2022). Past studies explored the link between green innovation (GI) and company growth, and the effects of increased market performance (MRP) and new market innovation (MRI) are often emphasized (Chandra et al., 2021). The link between small and medium-sized enterprises (SMEs) and more influential organizations is more vital than that between major corporations. Due to their nature and employment size, SMEs play a critical role in the current marketplace. Due to their ability to innovate new items and procedures has a tremendous effect on the economies of countries in an international market that is becoming more competitive (Padilla-Lozano and Collazzo, 2022). Consequently, enhancing the innovative abilities and expertise of SMEs opens immense opportunities. GIC is crucial for competitiveness and provides extra advantages for the private sector’s productivity and MRP (Khan et al., 2021).

In the current market of China, it is evident that the significance of SMEs has been expanding. The majority of China’s SMEs were founded within the previous three decades. Following China’s opening to the market economy in the 1980s as part of Deng Xiaoping’s market-oriented reforms, private SMEs were finally regarded as crucial to the nation’s economic growth (Fang et al., 2022). Large state-owned enterprises (SOEs) in China rapidly changed into small and medium non-SOEs due to the economic dynamics that influenced them till the end of 2004. Moreover, implementing a non-SOE marketing approach assisted in the growth of more SME businesses. China’s economic growth is increasingly dependent on the development of SMEs. Approximately 99 percent of all companies are SMEs, which significantly enhance a country’s economic growth and aid in expanding trade, commerce, and employment (Sharma et al., 2021). Considering this, SMEs remain confronted with numerous barriers to pursuing successful GIC. To enhance the MRP of SMEs, it is crucial to understand the factors influencing GIC (Yang, 2019). This research examines the relationship between GIC and MRP by employing the resource-based view (RBV) theory from a Chinese perspective.

To date, extensive research has concentrated on examining the relationship under the following domains. Although SMEs seem to be a trendy subject today, limited research studies have examined this phenomenon from emerging countries’ perspectives (Awan et al., 2022). For instance (Muisyo et al., 2022) explored the relationship between GI and environmental performance with green transformational leadership and green human resource management. Similarly, Arici and Uysal (2022) highlighted the association between GI, leadership, and green creativity. Despite previous studies, this study emphasizes GIC as a success element for SMEs MRP (Li et al., 2020). This study aims to comprehensively understand GIC paradigms and how they enhance the MRP of Chinese SMEs.

This study has significant contributions, which are as follows. The first contribution is to underline the significance of GIC and MRI to product innovation (PRI) in SMEs. It is essential to remember that GIC is required throughout all stages of rivalry and it creates wealth in the business sector (Rubio-Andrés et al., 2022). Numerous studies show that SMEs invest mainly in process development instead of PRI. Consequently, this study focuses only on the influence of PRI on MRP (Gherghina and von dem Berge, 2018). According to erstwhile research, a GIC must be formed, sustained, and fostered if organizations are competitive and produce new products (Waqas et al., 2021). Although the research concentrates on MRI and GIC, the significance of GIC and the effect of MRI on PRI were not well addressed in the prior study (Sprong et al., 2021). Secondly, this research investigates the significance of MRI techniques and PRI in achieving sustainable MRP. This study’s core argument is that MRI is essential when seeking to enhance MRP (Hussain et al., 2020). Marketing and PRI initiatives are significant components of MRI. Competitiveness has evolved into a critical element of market existence, but GIC initiatives produce more value and benefits, such as assisting a business to distinguish out from its rivals (Knut Haanaes, 2016). Lastly, this study is a pioneer because no previous study has been conducted in Chinese settings. Moreover, the emphasis of this research is to analyze the impact of innovative initiatives, such as GIC, on MRP of Chinese SMEs. This study adds to the existing body of knowledge by highlighting how effective MRP of SMEs can be achieved by fostering a unique relationship among GIC, PRI, and MRI under RBV theory (Hermundsdottir and Aspelund, 2021).

The rest of the research is organized as follows: The second section describes the literature review and formulation of hypotheses. The third section describes the methodology and research design. The fourth section contains findings and analysis. Section 5 concludes with a discussion of research results and policy implications, identification of study limitations, and recommendations for further research.

## Review of literature and development of hypotheses

As stated earlier, GIC leads to the invention of a novel technique, whether the organization is tangible (e.g., the manufacturing of a fresh commodity) or intangible (Granstrand and Holgersson, 2020; Chunxiang et al., 2022). Alternates to the existing business method are necessary for developing and discovering sustainable manufacturing and living structures. A significant amount of study has been shown on PRI, its function within SMEs, and MRI due to the importance of GIC in corporate growth and development (Afriyie et al., 2019; Castillo-Vergara and García-Pérez-de-Lema, 2021). Several scholars say several critical factors determine SMEs’ effectiveness. As per the cross-national study conducted by Wang and Juo (2021), robust marketing strategies, solid customer relationships, and competent leadership are all factors that lead to the SMEs efficient MRP (Castillo-Vergara and García-Pérez-de-Lema, 2021).

Al-Khatib (2022) explored seven substantial factors that contributed to the progress of SMEs, such as the ability to establish and maintain a technical advantage, the ability to identify and focus entirely on market segments, strong management, a significant “individuals interacting” framework, a competent customers’ business relationship, and the strategic utilization of information systems. Capable leaders, solid client and customer connectedness, an encouraging and robust control system, marketing effectiveness, establishing and keeping skills, and the right approach are six crucial elements that influence the MRP of SMEs (El Baz et al., 2022). Although most SMEs can swiftly adjust to changing surroundings and fulfill shifting consumer demands by adopting GIC (Adam and Alarifi, 2021).

Consequently, investigations usually emphasize the factors that adversely impact or impede the market performance of SMEs. However, according to the research conducted in Korea (Akbari et al., 2022), uncertain acceptance, risk-taking, ecologic strategic plan and monitoring, heterogeneous organizational nature, and professional competence in all workplaces are among the most distinguishing characteristics between innovative and non-innovative SMEs. It is believed that inadequate finance and poor rates of return are the most significant obstacles. Organizations that emphasize research and development (R&D), employee training, and personnel interaction are more likely to innovate. This scenario has led to increased new goods, technological advances in products and processes, and a greater focus on concept generation (Khan et al., 2022).

China, the country with the fastest-growing market worldwide, is one of the most significant places to investigate GIC and its accompanying phenomenon. The government classifies SMEs primarily by the number of workers, frequently fewer than 500 in most situations (Pan et al., 2021). The SMEs Promotion Law of China (2003) sets up the classification guidance for SMEs. The description of SMEs in Chinese settings is complicated since it depends on various elements, including business type, size, annual turnover, and net assets.

This research investigates the links between MRI, GIC, PRI, and MRP in Chinese SMEs. Consequently, the rationale for investigating the creative pursuits of Chinese SMEs is strengthened because several SMEs from developing nations indicate that GIC, PRI and MRI are essential for MRP. This study aims to find the critical building elements for improving a theoretical framework by analyzing studies on GIC in SMEs. A resource-based view (RBV) illustrates how internal resources impact MRP and enhance competitiveness in SMEs. RBV is a perspective that examines how high-performing companies allocate their attributes to their personnel. In addition, the RBV may assist in acquiring a greater knowledge of the success of these SMEs than other organizations.

According to most academics, GIC-related structures affect the SME’s MRP. This strategy was modified to accommodate the innovative character of SMEs from a creative standpoint. Several investigations used a variety of GIC-related structures as potential factors for model restriction. A business strategy, rivalry, technology, and culture are examples (Ali et al., 2021). On the other hand, innovation and marketing are essential to the success of many firms, as acknowledged in numerous management and marketing publications. Consequently, this research employs the GIC, MRI, and PRI criteria. The research framework is established by dividing GIC-related aspects into three main groups and studying how these elements affect the SMEs MRP.

### Market performance

SMEs’ personel, assets, and income all drop below the specific level. The definition of SMEs varies by nation and, in certain instances, by business type (Li et al., 2022; Ullah et al., 2022). In China, the purpose of SMEs is highly complicated as it appears that there is no one criterion. There are precise regulations on the overall assets of all manufacturing industry firms, such as those in gas, water, mining, construction, energy, and supply. Retail enterprises, transportation, hotels, and restaurants are considered SMEs; however, there are no asset restrictions. In contrast, the guidelines for the industrial sector for SMEs include no more than 2,000 employees and an annual turnover of a maximum of RMB 300 million. Their total assets must not exceed 400 million RMB (Zhang et al., 2022).

Operational distinctions between SMEs and large businesses have been widely studied previously. As described in the preceding paragraph, these distinctions develop concerning available resources and restrictions, ownership, decision-making, and the entire organization’s size. As in similar businesses globally, the absence of leadership, financial limits, and opposition to transformation usually fail too many SMEs (Lu et al., 2022). Due to these and various competitive market factors, SMEs must concentrate on productivity, innovation, and marketing. Even though China’s SMEs have overgrown over the past decade and have made significant contributions to the country’s development, their growth has been hampered by the aforementioned sluggish interconnections, lack of technical innovation, the market in general, and restricted financial assistance (Lu et al., 2022; Wang et al., 2022). This highlights the significance of effective market innovation while attempting to sell innovative products on both domestic and international marketplaces (Xu et al., 2020; Zheng C. et al., 2022). MRP leads to the link between sales drivers, market share, and product and service revenue premiums (Muisyo and Qin, 2021). Prior study has demonstrated a significant association between GI and a firm’s environmental performance. Whereas this study focused on the influence of GIC on SME MRP, relatively little attention was paid to the impact of GIC on SME MRP.

### Green innovation culture

GIC as a shared set of beliefs, concepts, and values produced by a management group to mold corporate behavioral patterns toward accomplishing shared objectives. GIC can be regarded as a systematic organizational culture that perceives environmental protection as foundational and a cornerstone of the company’s values (Chandra et al., 2021; Yao et al., 2021), assimilated into their mission statement in such a way that each team member in the firm internalizes a focus on environmental responsibility (Zheng et al., 2020; Khan et al., 2021). These GIC modifications play a crucial role in redefining the firm’s perspective towards environmental challenges, and workers now become more responsible about these matters. If managers care more about environmental preservation, the GIC will grow (Liu et al., 2022; Qu et al., 2022). GIC alters traditional modes of thought and acts as a catalyst for change (Waqas et al., 2021). Thus, a GIC may play a crucial role in engaging the organization’s workers in a more serious approach to environmental challenges (Zhu et al., 2022).

The formal structure of a GIC based on “eco-environmental ideals” may offer a company with essential insights for implementing environmentally friendly improvements in its operations (Wang and Juo, 2021). An organization’s pro-environment policy may be translated into GI *via* its GIC. However, GIC is only effective if a company can address environmental concerns (Aastvedt et al., 2021). Being the world’s biggest developing economy, China has become a global center for SMEs due to its GIC. China’s GI falls into two distinct types. The Chinese government is significantly responsible for technological innovation due to its assistance of businesses *via* encouraging and facilitating laws. This includes programs such as deep-sea space exploration and quantum computing breakthroughs. A further thread is a commercial innovation enabled by technology (Wang M. et al., 2021). Fewer restrictions to transformation and a high rate of entrepreneurial activity seem to be very probable in the nation, adding to its GIC. Consequently, Chinese SMEs have devised new strategies for developing different channels and implementing new methods of marketing a product that consumers value (Rehman et al., 2021). Due to their enhanced GIC, SMEs may gain a competitive edge in boosting production and marketing techniques and achieving desired results. The evidence indicates a significant relationship between GIC, PRI and MRI. Thus, based on the above arguments, we hypothesized that;

*H1a:* GIC positively influences the MRI of SMEs.

*H1b:* GIC significantly affects the PRI of SMEs.

### Marketing innovation

MRI is the introduction of a unique marketing approach comprising significant alterations to designing products or packaging, product endorsements, promotion of the product, or price. Product, and process innovations are more successful than marketing innovations, indicating that MRI complements product and process innovations rather than replaces them. As a result, MRI has the potential to lower costs or increase consumers’ willingness to pay (Sharma et al., 2021). Modern MRI positively impacts sales and reduces costs, enhancing competitiveness. Consequently, MRI is defined as exploring creative and innovative solutions to issues and needs (Chouaibi et al., 2022).

PRI is essential in MRI since it draws new consumers with the prospect of novel and improved products and expands product lines and segmentation. Consequently, MRI and PRI are often associated with a favorable connection. Due to the intense internal and worldwide rivalry, Chinese SMEs are noted for their MRI strategies (Darwish et al., 2021). Due to the enormous variety and diversity of accessible items, businesses must always create novel and improved marketing strategies to distinguish and advertise their items. This enables an inexpensive and lower-quality item and expands its distribution. Likewise, building a robust connection between MRP and MRI is uncomplicated since MRI leads to recruiting new clients and retaining the attention of existing ones, therefore favorably affecting MRP. Thus, we established the following hypothesis:

*H2a:* MRI positively influences the PRI of SMEs.

*H2b:* MRI significantly influences SMEs’ MRP.

### Product innovation

Although GIC may be seen in various ways, the emphasis of this section of the article will be on PRI. PRI is often connected with introducing a novel and improved product in the market, considering the current and future demands of the existing customers. PRI endeavors are famous for the Chinese government’s ongoing emphasis on technological improvements in goods and investment in research and development (R&D) (Gürlek and Koseoglu, 2021). To ensure and execute PRI, Chinese SMEs use a variety of approaches and tactics comprising process, cost, and technical innovation. China has generated a relatively limited number of unique product innovations from a global perspective. On the other hand, Chinese businesses are transitioning from progressive to dramatic breakthroughs due to their vast expertise with gradual developments. For instance, Sanyi Heavy Industry produces one of the most robust crawler cranes worldwide (Singh et al., 2022). Furthermore, to investigate this relationship, we develop the following hypothesis:

*H3:* PRI significantly influences the SME’s MRP.

## Methodology

### Development of the questionnaire

This research investigates four factors: GIC, PRI, MRI, and MRP among SMEs. All the factors were derived from earlier studies. The MRI items are taken from Padilla-Lozano and Collazzo (2022). The GIC items were taken from Gupta et al. (2016). The items of PRI were derived from Al-Abdallah and Al-Salim (2021). The items of MRP were taken from Mehralian (2022). A 5-point Likert scale was utilized to assess all items in which 1 represents strongly disagree, and 5 describes strongly agree.

### Sample and procedure

Primary data was gathered through an online questionnaire survey to investigate the hypotheses mentioned above. The participants of this study are managers, owners, salespeople, and R&D directors from SMEs working in China who are responsible for implementing innovative business strategies. Six hundred sixty-eight persons from various SMEs were asked to participate in the survey; 564 valid and complete questionnaires were received, with a 84.4 percent response rate. All survey respondents were briefed on the purpose of the investigation. Thity-five percent of the sample comprised company owners, whereas 27% had no more than 5 years of experience. Approximately 75% of the enterprises surveyed are small businesses with 1 to 50 workers (the number of working people).

There are various reasons to focus specifically on SMEs. Firstly, they contribute significantly to international economic growth and wealth creation. In addition, SMEs foster the development of jobs, leading to the most positive atmosphere in expanding marketplaces. Ultimately, innovative initiatives provide SMEs with the requisite skills to shorten production cycles, increase their probability of survival, and compete in fierce competition.

### Data analysis

AMOS (version 26) and SPSS (version 26) software packages are utilized to conduct statistical analysis. Structural equation modelling (SEM) is performed to examine the proposed hypotheses. SEM is a practical method for finding the connection between diverse variables that delivers reliable and valuable results (Steenkamp and Baumgartner, 2000) And has three significant benefits over older techniques. (i) A precise estimation of measurement error. (ii) The estimation of latent variables using observable variables. (iii) Verification of the model for evaluating and executing a pattern based on data compliance (Tanveer et al., 2021). In addition, the majority of multivariate techniques ignore computation error implicitly. Nevertheless, the SEM evaluates dependent and independent variables by accounting for computation error (Sardeshmukh and Vandenberg, 2013). Due to its reliability and robustness, the method yields precise and clear outcomes (Belaïd, 2017).

SEM enables the development of distinct indicator constructs per component and generates reasonable conclusions (Irfan and Ahmad, 2022). In addition, the error sections of the tested factors are measured. Therefore, the relationship between variables yields reliable results. In addition, it can examine complicated linkages and several hypotheses by integrating mean configurations and group evaluations, which other models and prototypes cannot do. Considering the benefits of SEM, we applied it in our research, as it is the most effective method for testing the link between all variables under investigation (see Table 1).

**Table 1:** Sample characteristics.

Characteristics	Frequency
*Position within the company*	
Business owner	35
Other	22
Board of directors	24
HRM Manager	11
Marketing Manager	4
R&D Manager	4
*Work experience*	
Less than 5 years	27
6–10 years	22
11–15 years	23
6–20 years	11
21–25 years	7
More than 25 years	10
*Organization’s size*	
Less than 10 employees	46
10–30 employees	13
31–50 employees	16
51–100 employees	7
100–300 employees	10
More than 300 employees	8

## Results

### Discriminant validity and correlation analysis

During the investigation, significant correlations between the variables were identified. The average variance extracted (AVE) square root was utilized to test the discriminant validity. AVE’s square root is more significant than its correlation with other factors, supporting the findings’ discriminant validity (Nureen et al., 2022). Comparing the AVE values to the maximum shared variance (MSV) values for each construct is an additional way to establish discriminant validity. If the AVE value for a particular construct exceeds the MSV value, discriminant validity is achieved (Fornell and Larcker, 1981). Our results corroborate this since every AVE value exceeds every MSV value. A convergent validity test was undertaken using item loadings and AVE to determine how strongly the items may be related (K.K., 2013). The data revealed that the variances of the latent variables remained larger than 50%, suggesting that the AVE values for each construct surpassed 0.50 (see Table 2).

**Table 2:** Descriptive statistics and correlations.

Variable	GIC	PRI	MRI	MRP	AVE	MSV
GIC	**0.744**				0.553	0.551
PRI	0.230	**0.842**			0.708	0.123
MRI	0.742	0.221	**0.779**		0.607	0.551
MRP	0.297	0.351	0.177	**0.715**	0.511	0.123

Bold values is square root of AVEs.

### Reliability analysis

The reliability of the item was evaluated using Cronbach’s alpha. The reliability of the data was validated by Cronbach’s alpha values that exceeded the suggested minimum level of 0.70 (Nunnally, 1978). Using the composite reliability (CR) technique, the item’s consistency across all variables was examined. Results indicate that CR values above the minimum threshold value of 0.70. CR levels exceed the minimum permitted threshold of 0.70 (Joe et al., 2017). Consequently, the results are shown in Table 3.

**Table 3:** Factor loading and discriminant validity.

Variable	Items	Standard loadings	Cronbach- α	CR
Green innovation culture			0.902	0.832
	GIC 1	0.782		
	GIC 2	0.825		
	GIC 3	0.879		
	GIC 4	0.865		
	GIC 5	0.854		
Product innovation			0.851	0.924
	PRI 1	0.763		
	PRI 2	0.828		
	PRI 3	0.764		
	PRI 4	0.808		
		0.728		
Marketing innovation			0.924	0.885
	MRI 1	0.754		
	MRI 2	0.726		
	MRI 3	0.740		
	MRI 4	0.754		
Market performance			0.916	0.807
	MRP 1	0.724		
	MRP 2	0.744		
	MRP 3	0.686		
	MRP 4	0.704		

CMIN/DF: 2.358; GFI: 0.967; CFI: 0.972; NFI: 0.963; IFI: 0.978; RMSEA: 0.049; AGFI: 0.962; RMR: 0.47.

### Structural model and results of hypotheses

**Figure 1:**
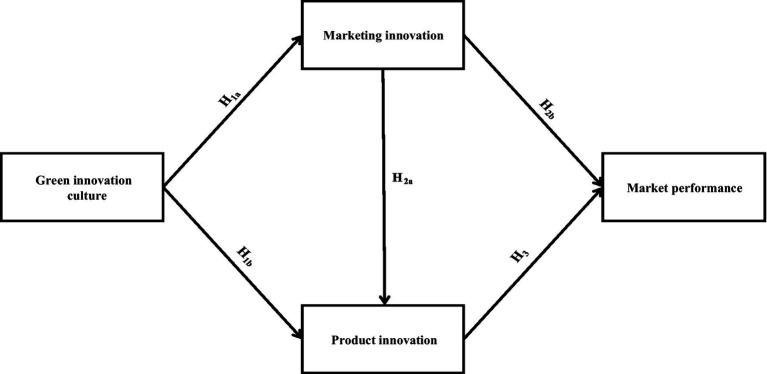
Depicts the study’s research framework.

**Figure 2:**
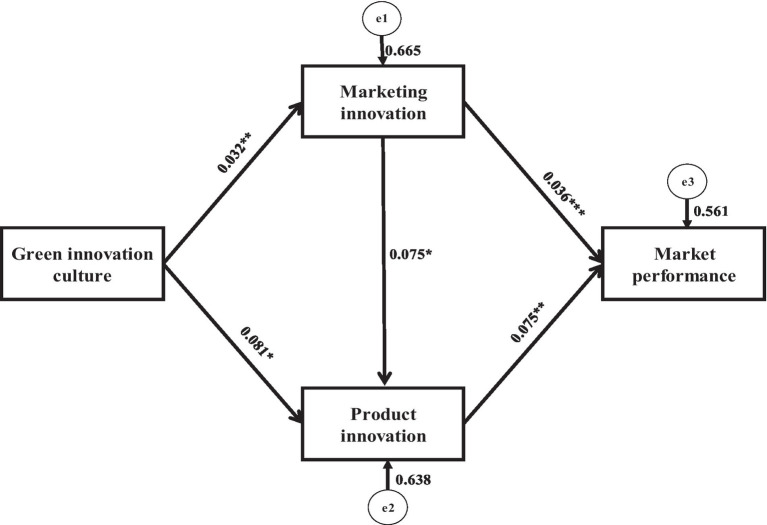
Path diagram of structural equation modeling. *** *p* < 0.01, ** *p* < 0.05, * *p* < 0.1.

We used SEM and covariance-based curve assessment techniques to examine the model’s connections. The study yielded a high *f*-value, suggesting that all links are linear. Several fitness tests were also conducted to guarantee that the data matched the structural framework (see Figure 1). The goodness-of-fit indices reveal that the data of this study is well-fitted. Figure 2 shows a path diagram of the SEM. A positive and significant correlation was found (β = 0.032, p 0.05) between the GIC and MRI. As a result, H1a is accepted. A significant association between GIC and PRI was established (β = 0.081, p 0.1). Hence, H1b was accepted. Likewise, a significant relationship between MRI and PRI was established (β = 0.075, *p* < 0.1). As a result, H2a was accepted. The MRI estimates (β = 0.036, p 0.01) reveal a substantial correlation with MRP, indicating that MRI favorably influences MRP. Consequently, H2c was confirmed. H3a was also approved, since PRI affects MRP strongly (β = 0.075, *p* < 0.05). The results of the hypothesis are shown in Table 4.

**Table 4:** The structural model.

Hypotheses	Hypotheses paths	*β*-value	*f*-value	Result
H1a	GIC → MRI	0.032^**^	148.2^***^	Accepted
H1b	GIC → PRI	0.081^*^	187.7^***^	Accepted
H2a	MRI → PRI	0.075^*^	242.6^***^	Accepted
H2b	MRI → MRP	0.036^***^	19.8^***^	Accepted
H3	PRI → MRP	0.075^**^	341.6^***^	Accepted

***p < 0.01, **p < 0.05, *p < 0.1.

## Discussions and policy implications

GIC seems to be the driving force behind attaining organizational objectives in the current competitive marketplace. In China’s SMEs, GIC looks crucial for developing R&D and implementing market strategy. Likewise, MRI knowledge may result in PRI and enhanced SMEs MRP. A corporation requires produced items to be efficient. Customers want a high-quality product at a low price, and future generations need a safe environment. Awareness of environmental issues has expanded due to the global climate catastrophe, which has led both consumers and governments to enact legal rules on this topic. Consequently, the accompanying pressure intensified market rivalry and prompted businesses to seek inventive solutions. The literature emphasizes that GIC gives a competitive advantage by enhancing the creation of new products and processes (Tariq et al., 2017). However, businesses confront risks such as ecological repercussions, laws, consumer expectations, and environmental uncertainty (Rayna and Striukova, 2019) while participating in GIC activities. Due to these risks, it may take businesses a considerable amount of time to get a return on their investments in GIC operations.

In addition to these factors, the conviction that GIC helps create environmentally friendly goods and processes and improves MRP inspired us to undertake this research. We analyzed the impact of GIC, MRI, and PRI on the MRP of Chinese SMEs. The findings showed that GIC could help SMEs to meet diverse customer needs, provides a unique product advantage, facilitates the development of new business models and better business opportunities, enhances the corporate image and improves MRP, and can help businesses gain a competitive advantage (Ahn et al., 2019), companies will benefit from investing in this direction.

### Theoretical implications

SMEs’ survival depends on their culture, capabilities, and values. This study discusses Chinese SMEs’ success in considering GIC, MRI, and PRI. The findings of this research are meant to contribute to the current literature on organizational dimensions, SMEs, and GI from a Chinese perspective. It significantly contributes to research on the SMEs MRP by exploring GIC frameworks in depth. This research model describes the relationship between SME GIC, PRI, MRI, and MRP. The findings indicate that GIC leads to MRI and PRI (H1a and H1b). Moreover, GIC is necessary for managerial, marketing, and organizational growth in competitive marketplaces.

GIC in Chinese SMEs had a favorable effect on their products’ marketing, R&D, and performance. When a company’s GIC is well-developed and diversified, it may not only foster the creation of creative concepts and goods but also devise marketing methods that capture customers’ attention. Additionally, GIC usually facilitates design and development procedures. According to the study, MRI shows a significant and positive connection with PRI and MRP (H2a and H2b). As per prior research, MRI significantly impacts MRP, firm profitability, and SMEs growth.

In contrast, this work improves previous studies by investigating MRI in an integrated approach focusing on SMEs MRP. When the creation and new product marketing are performed correctly, they are productive. Consumer awareness is lacking when a commodity is initially launched into the marketplace. Consequently, firms will want cutting-edge items to display and support them, culminating in MRI. Several studies have shown that PRI is crucial for the sustainable development of new goods, operational efficiencies, and market share expansion. This research shows a strong and substantial relationship between PRI and MRP (H3).

Moreover, the outcomes of this study indicate that GIC and MRI are closely related to PRI in SMEs settings. The research outcomes offer academics a valuable perspective, suggesting that GIC promotes SMEs to differentiate their goods from their rivals. This research contributes to the current GIC literature by enhancing knowledge of the relationship between GIC and the MRP of SMEs. Particularly, it investigates the impact of MRI and PRI on MRP.

### Managerial implications

This research examines the effects of PRI, MRI methods, and market expansion on the administrators of SME organizations. Initially, SMEs should make efforts on their MRI to gain a competitive advantage by fostering a GIC inside the firm. In the context of generating innovative and novel items, the management of SMEs must create new products and attain exceptional MRP. The research results also suggest that SMEs must attempt to maintain their assets to develop GIC, MRI, and innovative processes. These insights help managers to achieve improved MRP. SMEs must invest in promotional methods and build more substantial marketing initiatives throughout their firms to enhance their PRI skills. Furthermore, SMEs should be receptive to this GIC due to their technological environment and corporate branding activities, strengthening this ability to promote GIC for effective MRP.

As shown, the approach outlined in the research enables managers to adopt a new vantage point on how SMEs blend MRI and GIC to create effective MRP. Product and marketing development may flourish from incorporating a GIC into the firm’s structure. Therefore, managers may shape workers’ perceptions, attitudes, and acceptance of creative ideas to enhance MRP.

### Conclusion

This research employed the RBV theory to comprehend how GIC, PRI, and MRI may enhance the MRP of Chinese SMEs. The results demonstrate that managers in emerging economies must pay more attention to the GIC, MRI, and PRI. They must increase their GIC spending. This research reveals the relationship between GIC and emphasizes the significance of MRI and PRI for SMEs’ MRP. SEM tests have supported the link between GIC, MRI, PRI, and MRP. In addition, Chinese SMEs are less sophisticated and superior at embracing GIC than their counterparts in industrialized countries. Several Chinese SMEs are subject to stringent environmental restrictions imposed by the government and local and international consumers. Due to consumers’ belief that GIC is a fast answer to these issues, GIC has been employed primarily to meet customers’ desires, needs, and requirements. The developed countries, on the other hand, put a heavy focus on the GIC, PRI, and MRI because they recognize that without applying these factors, they would fail to achieve their intended objectives. They will not fulfill the client’s requirements.

In contrast to earlier studies, this study includes limitations that provide areas for further exploration. It was difficult to obtain data through direct surveys because of the COVID-19 epidemic. Thus, we employed social media or email for data gathering, and future studies on the targeted respondents may include direct surveys. Second, it is suggested that a random sample method be employed to collect data since the snowball technique used in this study offered a risk of brief demonstration and might influence the interpretation of findings. The generalization restriction is the third. This research is conducted in an emerging country (China). The GIC, MRI, PRI, and MRP may vary in industrial environments and be influenced by the unpredictability of environmental contingency. Future researchers must include other potential variables in the relationship between GI and MRP, such as corporate social responsibility, environmental strategy, and green intellectual capital.

## Data availability statement

The original contributions presented in the study are included in the article/supplementary material, further inquiries can be directed to the corresponding author.

## Ethics statement

This study was reviewed and approved by Ocean University of China (protocol code 704–3 on 13-09-2021). The patients/participants provided their written informed consent to participate in this study.

## Author contributions

CW: conceptualization and writing – original draft. XC: formal analysis, data handling. XS: variable construction, software, methodology, and writing – review and editing. All authors contributed to the article and approved the submitted version.

## Conflict of interest

The authors declare that the research was conducted in the absence of any commercial or financial relationships that could be construed as a potential conflict of interest.

## Publisher’s note

All claims expressed in this article are solely those of the authors and do not necessarily represent those of their affiliated organizations, or those of the publisher, the editors and the reviewers. Any product that may be evaluated in this article, or claim that may be made by its manufacturer, is not guaranteed or endorsed by the publisher.
